# Thin Modified Nitrided Layers of High-Speed Steels

**DOI:** 10.3390/ma18112434

**Published:** 2025-05-23

**Authors:** Khrystyna Berladir, Tetiana Hovorun, František Botko, Svetlana Radchenko, Oleksandr Oleshko

**Affiliations:** 1 Department of Applied Materials Science and Technology of Constructional Materials, Faculty of Technical Systems and Energy Efficient Technologies, Sumy State University, 116, Kharkivska St., 40007 Sumy, Ukraine; hovorun@pmtkm.sumdu.edu.ua; 2 Department of Automobile and Manufacturing Technologies, Faculty of Manufacturing Technologies, Technical University of Košice, Bayerova 1, 08001 Prešov, Slovakia; frantisek.botko@tuke.sk (F.B.); svetlana.radchenko@tuke.sk (S.R.); 3 Academic and Research Medical Institute, Sumy State University, 1, Sanatorna St., 40007 Sumy, Ukraine; o.oleshko@med.sumdu.edu.ua

**Keywords:** industrial growth, process innovation, AISI M2, AISI M41, high-speed steel, ion nitriding, microhardness, wear resistance

## Abstract

The study examined the influence of ion-plasma nitriding on the structure, mechanical, and tribological properties of high-speed steels AISI M2 and AISI M41. A comprehensive study was conducted on the changes in phase composition, microhardness, and wear resistance of the obtained modified layers. It was established that the optimal approach was the formation of high-nitrogen martensite without excessive nitrides, which ensured improved mechanical properties of the steels. The dependence of the nitrided layer depth and its microhardness on nitriding temperature and duration was investigated. It was found that at a temperature of 480–520 °C and a processing duration of up to 1 h, a hardened layer with a depth of 25–40 μm was formed, exhibiting increased wear resistance and microhardness of up to 10–12 GPa. The analysis of structural transformations confirmed the presence of ε and γ’ phases, which contributed to increased strength and reduced friction coefficient. The obtained results can be used to improve the technological processes of heat treatment for high-speed steels used in the production of cutting tools. The proposed nitriding parameters contribute to extending the service life of steel components, which is relevant for the mechanical engineering and metallurgical industries.

## 1. Introduction

Cutting processing is a complex and expensive process, which is characterized by significant workability and metal consumption [1,2]. For effective cutting processing, tool materials must have properties such as hardness, wear resistance, strength, thermal conductivity, heat resistance, and heat capacity. The most common material for the manufacture of cutting tools is high-speed steel (HSS), which combines a complex of performance properties such as high strength, wear resistance, hardness, heat resistance, and increased resistance to plastic deformation [3]. HSSs are widely used for cutting tools that operate under conditions of significant load and heating of the working edges. Tools made of HSSs have high stability of properties, which is especially important in conditions of flexible automated production [4]. The durability and technical-economic performance of machining processes depend on the wear resistance of the cutting tool. Therefore, in the development of new types of tools, special attention is given to enhancing their wear resistance and performance, which are determined at all stages of creation, from design to manufacturing technology and strengthening treatments.

For many steel products and parts to improve their operational properties, various coating technologies are widely used to increase wear resistance [5,6] and corrosion resistance [7], reduce friction [8], increase heat resistance and thermal stability [9], improve electrophysical properties [10], etc.

Currently, significant progress has been made in the development of technologies for applying wear-resistant coatings. However, there are still certain issues that can only be resolved through an in-depth study of the processes and patterns that occur in the contact zone between the cutting tool and the workpiece material. To increase the wear resistance of the cutting tools, thereby improving the machining process and achieving the desired surface quality of the workpiece, several measures can be proposed. These include changing the tool geometry, implementing a set of actions aimed at controlling feed rate and the presence of a support under the workpiece during processing, as well as surface modification methods such as applying coatings to the cutting edges of the tool. The use of wear-resistant coatings makes it possible to control the properties of tool materials, tool wear, and the performance of cutting tools from HSS by selecting the appropriate composition, structure, and design of the coating, as well as the technological conditions of its application [11].

One of the progressive methods to achieve this goal is ion-plasma nitriding, which allows the formation of thin modified layers on the surface of steel to improve its properties [12,13,14]. The procedure involves bombarding the metal surface with nitrogen ions, which leads to the formation of a nitrogen-containing layer on the surface of the part. Nitrogen ions, typically in molecular form, reach the metal surface under the influence of high temperature and vacuum. In practice, ion nitriding is used for machine parts where surface wear is the primary cause of failure. This process increases the wear resistance and service life of metal components, especially in industries such as automotive, aerospace, and tool manufacturing. The successful application of ion nitriding in various industries confirms its importance in its ability to form hard, thin nitride layers and improve tool properties without altering their overall structure or shape, thus preserving the dimensional accuracy and integrity of HSS components.

The advantages of ion-plasma nitriding include the following [15,16,17]:

- the ability to control the saturation process, which ensures the production of a high-quality coating with a given phase composition and structure;
- ensuring identical activity of the gas environment over the entire surface of the part covered by the glow discharge; this ultimately ensures the production of a nitrided layer of uniform thickness;
- reducing the complexity of local protection of surfaces that are not subject to nitriding (using metal screens);
- a sharp reduction in the duration of nitrided parts (by 2–2.5 times);
- reducing deformation of parts.

The use of ion-plasma nitriding instead of carburizing, nitrocarburizing, gas or liquid nitriding, and bulk or high-frequency hardening allows for saving on basic equipment and production space, reducing machine tool and transportation costs and reducing the consumption of electricity and active gas media [18,19].

In ion-plasma nitriding, diffusion saturation of the surface with nitrogen occurs using low-temperature plasma [20]. The method of diffusion saturation of the surface with nitrogen using low-temperature plasma qualitatively differs from classical schemes of chemical-thermal treatment in a significantly higher diffusion rate, the possibility of reducing the process temperature, and environmental friendliness. During the saturation process under the influence of ion bombardment, an increased concentration of structural defects occurs in the steel, which contributes to an increase in the intensity of diffusion processes, including nitrogen mass transfer, and creates the possibility for the appearance of new phases that are not formed under normal saturation conditions. One of the obstacles to the widespread introduction of the ion-plasma nitriding method into industry is the lack of data for optimizing technological regimes [21]. In this regard, determining the effectiveness of using this type of nitriding for different types of steel and product operating modes is an urgent task.

In [22], it was shown that ion nitriding did not provide surface hardening of the material in the holes due to the occurrence of the hollow cathode effect. The works [23,24] presented the results of nitriding with a hollow cathode of the surface of small-sized parts made of martensitic stainless steel AISI 420, which resulted in a surface layer 20 μm thick with a steel microhardness of more than HV 1000. After such treatment of steels X6CrNiTi1810 and X2CrNiMoN2253 stainless steel plates, a layer 80 μm thick had a microhardness on the surface of more than HV 1400 [25].

Ion-plasma nitriding in gas plasma of vacuum-arc installations at temperatures from 500 to 600 °C not only allowed to reduce nitriding time to 1 h but also, in comparison with furnace nitriding, increased the relative wear resistance of the nitrided layer [26,27]. Also made it possible to control the phase composition (by changing the energy characteristics of the process or the ratio of working gases in a mixture of nitrogen and argon) and created the possibility for the emergence of new phases based on nitrogen compounds [28,29,30]. Thus, in study [28], the formation of a metastable nitrogen-rich solid solution phase in austenitic chromium-containing stainless steels was discussed. This phase formed due to the high nitrogen content and specific implantation conditions. It possessed unique properties, including increased hardness, magnetic characteristics, and altered nitrogen diffusion behavior. The authors of [29] examined an extremely relevant and technically significant topic. Duplex surface systems opened new possibilities for improving the mechanical properties of tool materials, especially those operating under high-load conditions. The study suggested that both approaches, ex-situ plasma nitriding prior to PVD coating and in-situ HPMFPPN followed by CrAlN deposition, have their advantages. Ex-situ nitriding allows for precise control of substrate strengthening but requires separate production stages, whereas in-situ HPMFPPN is more economical in terms of integration into the manufacturing process, although its control can be more complex. In study [30], an analysis was conducted on the potential structural changes determined by non-equilibrium processes in vacuum-plasma coating methods. It was shown that the non-equilibrium conditions of ion-plasma deposition significantly expand the range of possible structural states of the formed material, from amorphous to highly ordered crystalline, due to the dynamics of structural transformations in vacuum-plasma coatings. These non-equilibrium deposition conditions indeed play a key role in forming the microstructure of coatings, enabling the creation of unique material states that cannot be achieved through equilibrium methods. The ability to modify the lattice structure by controlling the parameters of the ion-plasma flow opens prospects for precise control over mechanical and tribological properties. These new phases were not formed under conditions of conventional nitrogen saturation at elevated temperature [31,32,33] and also allowed getting rid of the harmful effects of ammonia.

In the process of saturation under the action of ion bombardment, the concentration of structural defects in the material significantly increased [34], which contributed to an increase in the intensity of diffusion processes, including nitrogen mass transfer [35]. Such nitriding was usually carried out after thermal and mechanical treatments. In this case, during the nitriding process, a negative potential was applied to the products, adjusting their value in such a way as to maintain the temperature of the products in the range from 500 to 600 °C. The surface hardness of the products after nitriding was high and is up to 11–17 GPa, depending on the steel composition [36]. Comparison of the research results with similar studies in other areas of surface treatment and steel modification can help determine the competitiveness and advantages of this method [37].

Thus, plasma nitriding methods ensure the formation of a nitrided layer with a desired structure on the surface of treated parts. This improves the wear resistance and thermal stability of the tool. The nitrided surface of the tool, with its reduced friction coefficient and enhanced anti-friction properties, facilitates easier chip removal and prevents chip adhesion to the cutting edges, as well as the formation of wear pits, which allows for increased feed rates and cutting speeds [27,36]. As a result of ion nitriding for cutting tools made from HSSs (milling cutters, drills, taps, dies, etc.), their cutting properties were improved, and wear resistance and productivity were increased [27].

The aim of this study was to investigate the effect of ion-plasma nitriding on the structure, microhardness, and wear resistance of high-speed steels AISI M2 and AISI M41 to improve the quality and service life of tools made from these materials. To achieve the goal of the study, firstly, the processes of formation of thin modified layers in steels AISI M2 and AISI M41 during ion-plasma nitriding were studied. Secondly, the analysis of structural-phase transformations in steels during ion-plasma nitriding was carried out. Finally, the influence of the structural-phase state of modified layers on the mechanical and tribological properties of steels AISI M2 and AISI M41 was assessed. This study provided new insights into the mechanisms of diffusion-controlled structural-phase transformations in materials subjected to ion-plasma nitriding, particularly the effect on tool HSSs.

## 2. Materials and Methods

### 2.1. Materials

The work conducted research for steel AISI M2 (T11302, DIN 1.3243, EN 1.3339, Ukrainian analog R6M5) (NICMAS, Sumy, Ukraine) and steel AISI M41 (T11341, DIN 1.3343, EN 1.3243, Ukrainian analog R6M5K5) (NICMAS, Sumy, Ukraine). It is possible to make almost any type of cutting tool from steel AISI M2, which is used for processing steels, non-ferrous metals, alloys, cast iron, wood, composite, and other materials. Instead, steel AISI M41 is used for processing high-strength stainless and heat-resistant steels and alloys in conditions of increased heating of the cutting edge.

The chemical composition of the proposed steels is given in Table 1, and the main characteristics of the studied HSSs in the as-delivered condition are given in Table 2.

The study of the effect of ion-plasma nitriding on the structure and properties of HSSs was carried out after standard heat treatment, which included isothermal annealing in the temperature range of 840–870 °C, cooling to 740–750 °C, holding for at least 1–1.5 h in a furnace, cooling to 600 °C in a furnace and then in air, quenching with heating in the temperature range of 1200–1230 °C, cooling in oil, 2-fold tempering at 540–560 °C, cooling in air (obtained hardness HRC 63–65).

### 2.2. Ion-Plasma Nitriding Process

**Figure 1:**
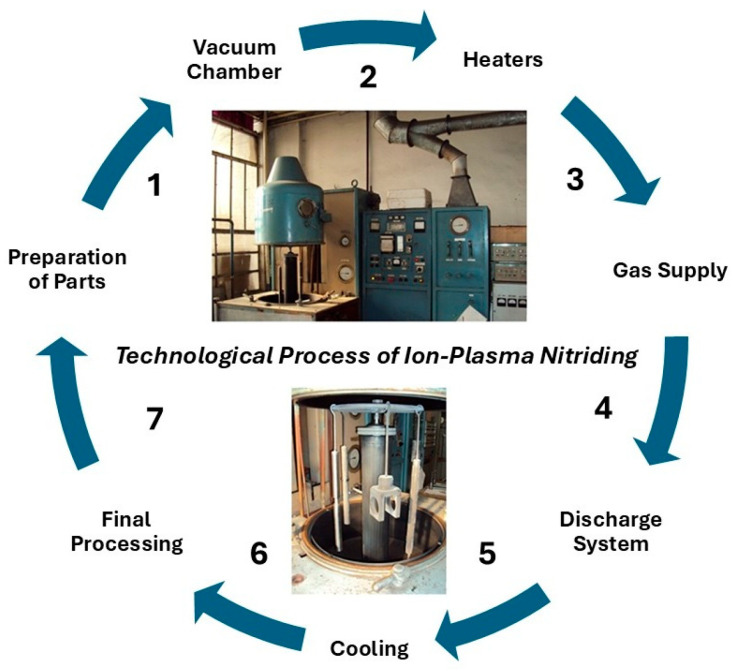
Realization of the technological process of ion-plasma nitriding (The numbers show the sequence of stages of the technological process).

The technological process of ion-plasma nitriding is presented in Figure 1.

It involves the following sequence of operations: the tools to be coated with nitride layers are placed in the chamber of the installation and connected to the negative electrode. The chamber is then sealed by evacuating air to a pressure of 1.4 × 10^2^ Pa and purged with working gas for 5–15 min at a pressure of 13 × 10^2^ Pa. After that, the chamber is evacuated again to a pressure of 26–52 Pa, voltage is applied to the electrodes, and a glow discharge is initiated. After surface treatment for 30–60 min, the voltage is reduced from 1100–1400 V to the operating level (200–500 V), and the pressure is increased to (1.3–13) × 10^2^ Pa. The working process temperature (350–570 °C) is reached within 15–30 min. The heating rate depends on the surface area and mass of the parts. The ion-plasma nitriding temperature varied between 350 and 570 °C, depending on the experimental conditions, and the holding time also ranged from 30 to 60 min. These studies were conducted to determine the optimal conditions for obtaining the best coating properties for high-speed steels AISI M2 and AISI M41. After ion-plasma nitriding, the parts were cooled to room temperature in a vacuum. These basic steps of the ion plasma nitriding process allow for improving metal products, giving them higher strength, corrosion resistance, and wear resistance.

Nitriding was carried out in the NGV-6.6/6-I1 electric furnace (NICMAS, Sumy, Ukraine). Its design and operating principle of the installation were described in detail in the previous work [38].

### 2.3. Methods

Metallographic analysis was performed using an optical light microscope MIM-7 (“Asma-Prylad, Ltd.”, Svitlovodsk, Ukraine). A 4% alcoholic solution of nitric acid was used for etching metallographically prepared surfaces of steel samples.

To measure the microhardness of steel samples, a PMT-3 device (“Standard-M”, Zaporizhzhia, Ukraine) was used, with loads on the indenter P = 1 N and holding at this load for 10 s [38]. Hardness measurements were performed on cross-sectional samples.

The phase composition and structural state were investigated by X-ray diffraction on a DRON-3M diffractometer (INTERROENTGEN, Kharkiv, Ukraine), the principle of which is based on the phenomenon of X-ray diffraction during layer-by-layer analysis of the sample surface in copper Kα1 radiation. The shooting was carried out in the spot mode with a scanning step Δ(2θ) = 0.05–0.2° and a pulse accumulation duration at each point of 20–40 s. To remove surface layers during X-ray diffraction depth profiling, a layer-by-layer etching method with a 4% alcoholic solution of nitric acid (chemical etching method) was used.

Wear resistance tests under sliding friction conditions were performed on the SMC-2 machine (NICMAS, Sumy, Ukraine) using the “roller-roller” scheme, the test methodology of which is standardized. The principle of operation of the machine was to abrade a pair of samples pressed against each other with force. In our case, one roller rotated, and the other was stationary for the simulation of sliding friction under different conditions. In all cases, steel AISI M2 heat-treated to a hardness of 63–65 HRC were used as the counter-body material. The wear testing results were conducted under fixed testing conditions. The sliding speed was 1–4 m/s, load 1–4 MPa, friction distance 50–200 m. After the tests, the samples were washed with alcohol, dried, and weighed on electronic scales VA 200 with an accuracy of four decimal places at a temperature of 20 °C. The friction coefficient was determined during the first 200 s of friction (at the time of running-in), and the average value was calculated.

### 2.4. Criteria for Selection and Evaluation of Coatings

The study of criteria for selecting and evaluating coatings for analyzing the formation of thin modified layers in steels during ion-plasma nitriding aimed to develop a system for assessing the efficiency and quality of the resulting coatings, as well as to establish key criteria that will allow choosing the optimal process parameters to achieve the required characteristics of high-speed steels.

The optimal structure of nitrided HSS is high-nitrogen martensite that does not contain excessive nitrides [35,36,37]. This structure was achieved by saturating the tool surface with nitrogen at a temperature of 480–520 °C during short-term nitriding (up to 1 h).

The study of the patterns and mechanisms of diffusion-controlled structural-phase transformations in steels during low-temperature ion-plasma nitriding (at temperatures below 550 °C) involved analyzing nitrogen diffusion processes, phase transformations in the material, and examining the impact of these transformations on the mechanical and tribological properties of steel AISI M2 and AISI M41.

The obtained results can be used to improve technological processes and develop new materials with enhanced characteristics for specific industrial applications (Table 3). These criteria are recommended guidelines and may vary depending on the specific parameters of the nitriding process, the quality of the starting material, and the requirements for the end use of high-speed steels. For specific research and production tasks, it is important to establish precise evaluation parameters based on requirements and specifications.

In connection with the study of the patterns and mechanisms of thin-modified layer formation in steels during ion-plasma nitriding, it was essential to analyze the influence of various process parameters on the structural and physicochemical properties of the obtained layers. This included examining the effects of temperature, processing time, gas environment, nitrogen concentration, and other factors on the thickness, microstructure, phase composition, and mechanical characteristics of the coatings.

## 3. Results

### 3.1. Structure, Morphological Features, and X-Ray Diffraction Analysis of Nitrided Coatings

**Figure 2:**
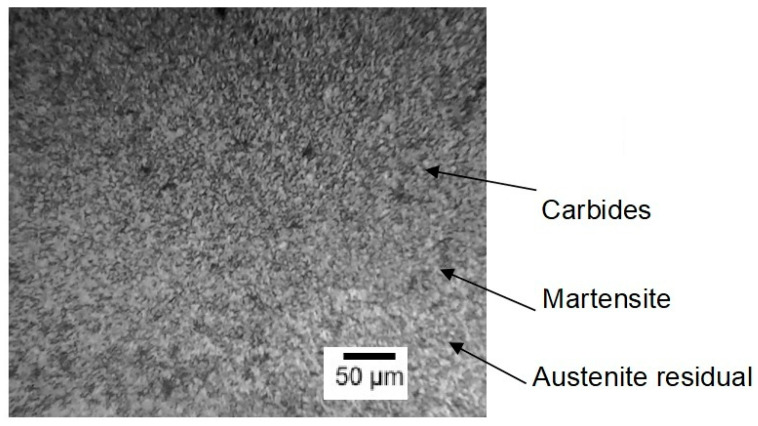
Microstructure of steel AISI M2 after standard heat treatment [39].

After properly conducting heat treatment, steels AISI M2 and AISI M41 should have a martensite structure with evenly distributed small and medium-sized carbides (Figure 2). In tempered martensite, fine dispersed carbides are uniformly distributed, formed from retained austenite and quenched martensite. Due to their high dispersion, it is difficult to determine their exact composition and structure, but they are special alloy carbides: M_6_C (a carbide based on tungsten and molybdenum), M_23_C_6_ (a chromium carbide), and MC (a vanadium carbide) in small quantities (M denotes the metal in the carbide). These carbides provide the steel with high hardness and thermal resistance, making it ideal for cutting tools.

**Figure 3:**
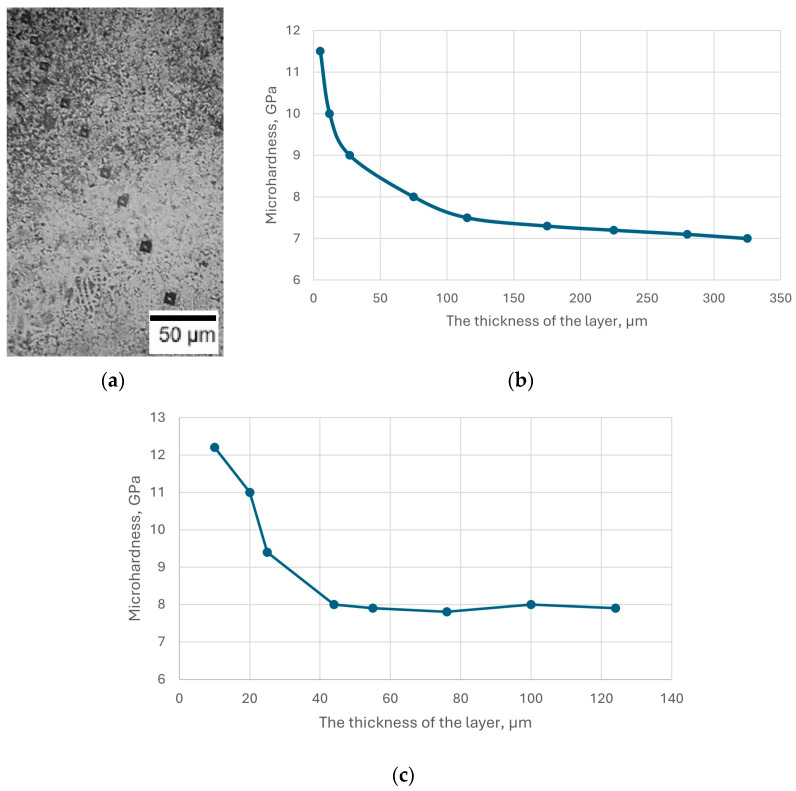
Image of indenter marks during hardness measurement on the surface of nitrided AISI M4 steel (**a**); change in hardness along the depth of the nitrided layer of AISI M41 (**b**) and AISI M2 (**c**) steels.

Microhardness measurements were performed on the cross-section of the samples, from the coated surface into the depth of the samples, to the core (uncoated base) (Figure 3). The microhardness of the nitrided layer of steel AISI M2 reached a maximum value of 10.5–11.5 GPa and then decreased to the initial value of the steel (Figure 3c). The high microhardness of the surface layer of steel AISI M2 can be explained by the formation of nitrogen martensite and dispersed nitride (phases ε and γ’), as well as the presence of high-hard nitrides and carbonitrides of alloying elements such as Mo, W, Cr, V. The structure of the nitrided surface layer, which has these characteristics, contributes to high wear resistance and improvement of the operational properties of steel. The optimal structure of the nitrided steel AISI M41 was high-nitrogen martensite, which did not contain excess nitrides. The specified structure was provided by saturating the surface of the tool from steel AISI M41 with nitrogen at a temperature of 480–520 °C in the process of short-term nitriding (up to 1 h). In this case, a hardened layer with a depth of 20–40 μm was formed with a surface microhardness of 10–12 GPa and a core hardness of 8–9 GPa (Figure 3b).

**Figure 4:**
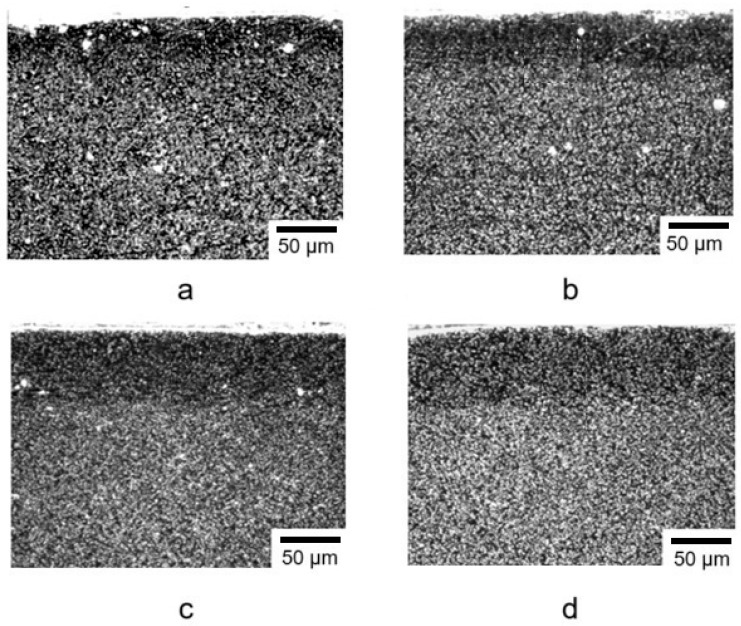
Microstructure of steel AISI M2 after ion nitriding at a temperature of 500 °C for different nitriding times: (**a**)—30 min; (**b**)—40 min; (**c**)—50 min; (**d**)—60 min.

The structure of steel AISI M2 after ion nitriding for different nitriding times is presented in Figure 4. The microstructure of the samples exhibited a dark diffusion zone, which represented nitrogenous ferrite with a BCC lattice. The transition from the nitrided layer to the base (matrix) of the material was gradual, which was one of the main requirements for the microstructure of nitrided steel and correlated with the data in [40].

The results of microhardness measurements at different nitriding times are summarized in Table 4.

**Figure 5:**
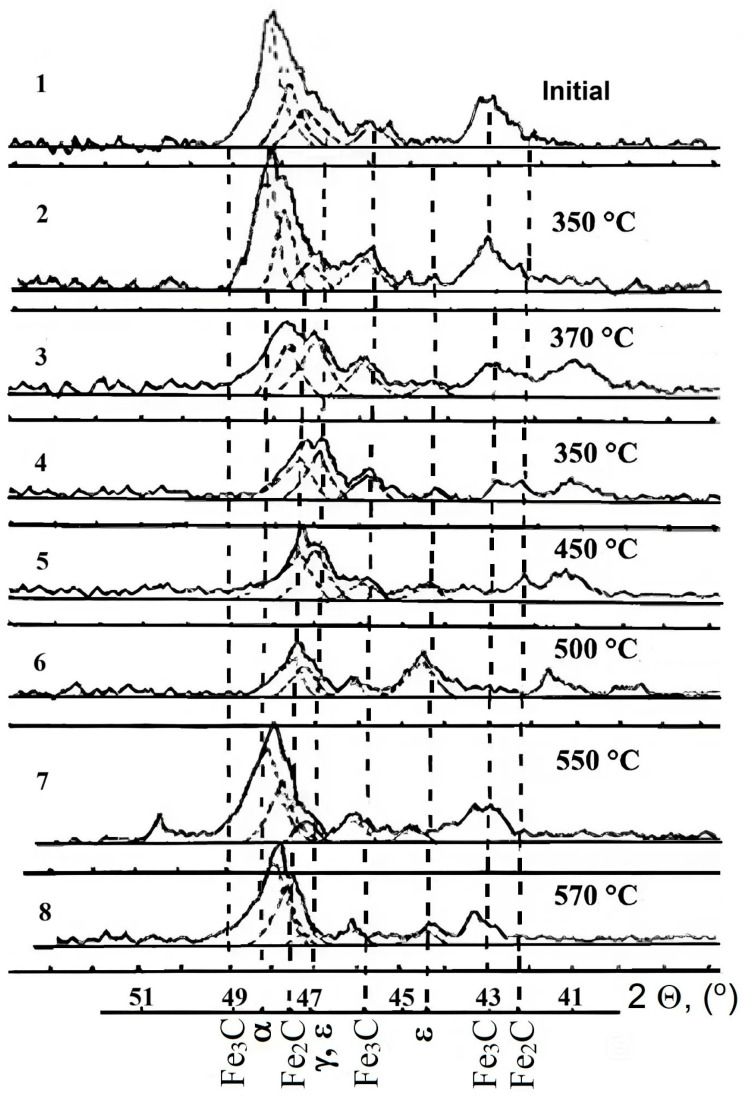
Fragments of diffraction patterns of steel AISI M2 nitriding for 40 min at different temperatures: 1—initial state; 2–8—treatment at temperatures of 350–570 °C; 2Ɵ—X-ray reflection angles (dotted lines indicate the formed phases).

**Figure 6:**
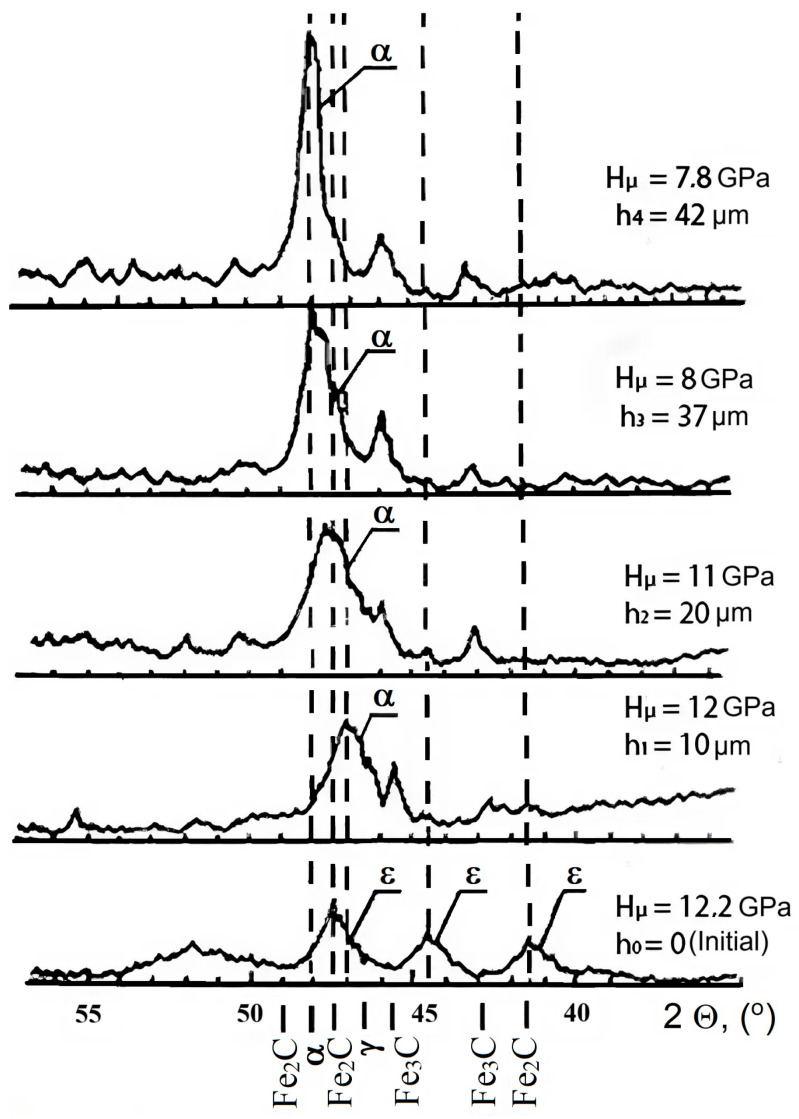
Fragments of diffraction patterns taken for nitrided steel AISI M2 at different depths (dotted lines indicate the formed phases).

Changes in the phase composition of the surface of steel AISI M2 are shown in Figure 5 and Figure 6. The comparative analysis of diffraction patterns showed that nitriding at P_N_ = 5 × 10⁻^2^ Pa and a temperature of 350 °C for 40 min led to significant changes in the phase and structural states. The shift of the diffraction maximum (110) Fe to the low-angle region indicated the formation of a nitrogen solid solution in iron and the appearance of Fe_2-3_N traces. This trend intensified with increasing nitriding temperature. For samples nitriding at temperatures of 450–500 °C, an ε-phase was observed at the X-ray penetration depth. At higher nitriding temperatures (550–570 °C), nitride phases were not formed in significant quantities on the surface, and the increase in microhardness was mainly due to the formation of a nitrogen solid solution in iron.

Layer-by-layer X-ray analysis provided a typical picture of phase composition changes with depth (Figure 6). In the near-surface zone, at an X-ray penetration depth of approximately 3 μm, a nitrided layer consisting of Fe_2-3_N and Fe_4_N was present. As the layers were removed, lower nitrides and a solid solution with different nitrogen concentrations remained. The diffraction maximum (110) shifted towards larger reflection angles and approached the value corresponding to the initial state of AISI M2 steel at a depth of ∼40 μm.

The main advantage of Ion nitriding of AISI M41 tool steel was the ability to obtain either a purely diffusion-strengthened layer or a mono-phase Fe_4_N (γ’) nitride layer on the surface, which corresponded with [41]. This contrasted with classical gas nitriding in ammonia, where the nitride layer consisted of two phases (γ’ + ε), which introduced internal stresses at the phase boundary, leading to brittleness and delamination of the hardened layer during operation.

The main alloying elements of steel AISI M41 are W, Mo, V, Co, and Cr. These elements form special carbides in the steel as M_6_C based on W and Mo, MC based on V and M_23_C_6_ based on Cr. X-ray structural analysis proved that the structure of the steel contained M_6_C and MC carbides, but M_23_C_6_ carbides were absent.

Some studies indicated that after standard heat treatment, the structure of M41 steel contains only M_6_C carbide particles [41]. This was apparently due to the small volume fraction of MC carbides and the similarity of these particles to the matrix, which cannot be detected.

Thus, research has shown that ion-plasma nitriding affected the structural properties of AISI M2 and AISI M41 steels. The duration of the isothermal holding process significantly influenced the depth and microhardness of the modified layer. Increasing the nitriding duration promoted the formation of a more durable and wear-resistant layer by altering the structural and phase properties of the steel. The analysis of the results revealed that the composition of alloying elements also changed during the nitriding process. This indicated a complex mechanism of nitrogen interaction with the steel surface and the formation of stable nitride phases.

### 3.2. Influence of Different Coating Process Parameters on Microhardness

The importance of controlling the duration and conditions of nitriding to achieve optimal material properties of high-speed steels is emphasized in these studies.

**Figure 7:**
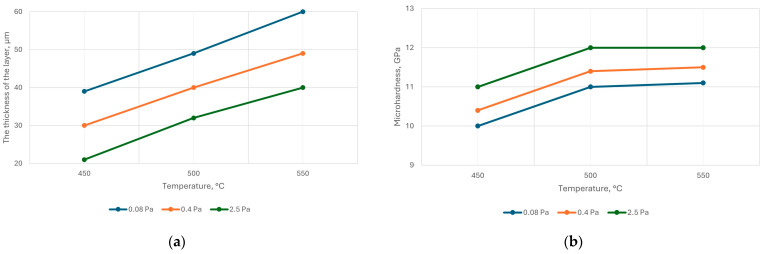
Dependence of the thickness (**a**) and microhardness (**b**) of the nitride layer of steel AISI M2 on the temperature at different nitrogen pressures; nitriding time 40 min.

Experiments to determine the dependence of the thickness and microhardness of the nitrided layer on temperature showed the following results (Figure 7).

The voltage on the samples was negative, and its value was regulated while maintaining the set temperature within 150–200 V. The graphs showed that all other conditions were equal; with increasing nitrogen pressure, the nitriding rate decreased, and the microhardness increased.

**Figure 8:**
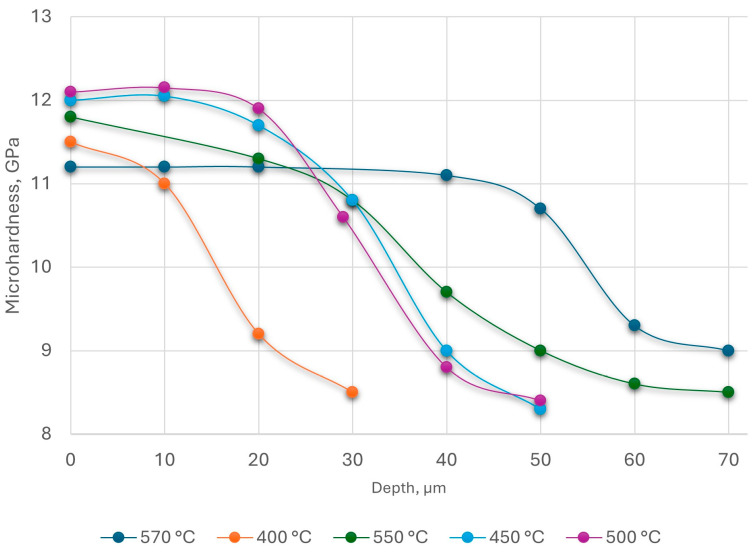
Distribution of microhardness by depth of steel AISI M2 at different temperatures: t = 40 min; P_N_ = 5 × 10^−2^ Pa.

The nature of the microhardness distribution over the thickness depending on the nitriding temperature is shown in Figure 8.

From Figure 8, the microhardness of the upper layers obtained at a temperature of 570 °C was lower than that of those nitrided at lower temperatures due to the decomposition of the ε-phase, which had higher hardness than the solid solution of nitrogen in iron.

**Figure 9:**
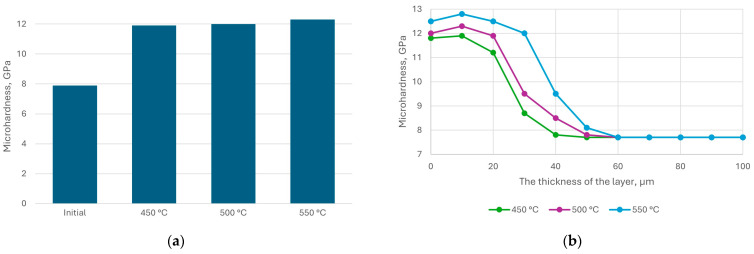
Distribution of microhardness of steel AISI M41 depending on nitriding temperature (**a**) and depth (**b**).

Figure 9 shows the dependence of the surface microhardness of steel AISI M41 on the nitriding temperature and the distribution of microhardness along the depth of the nitrided layer.

The microhardness of the surface after nitriding increased by (1.5–1.6) times. A significant increase in microhardness was observed near the surface of treated steel samples. The transition zone exhibited a smooth gradient from the hardened layer to the substrate, while the microhardness of the substrate itself remained largely unchanged. The maximum microhardness increased with nitriding temperature, which was attributed to the structural-phase state of the modified surface layer. The structural-phase state of the modified layer in steel AISI M41 changed depending on the nitriding temperature. At 500 °C and 550 °C, Fe_4_N (γ′-phase) formed on the surface alongside nitrogen-enriched martensite. However, at 550 °C, fine-dispersed particles with an average size of approximately 0.1 μm appeared on the steel surface.

Ion-plasma nitriding of steel AISI M41 drills led to a comprehensive improvement in the working surface properties, including increased hardness and wear resistance. In general, ion nitriding significantly extends the service life of drills.

**Figure 10:**
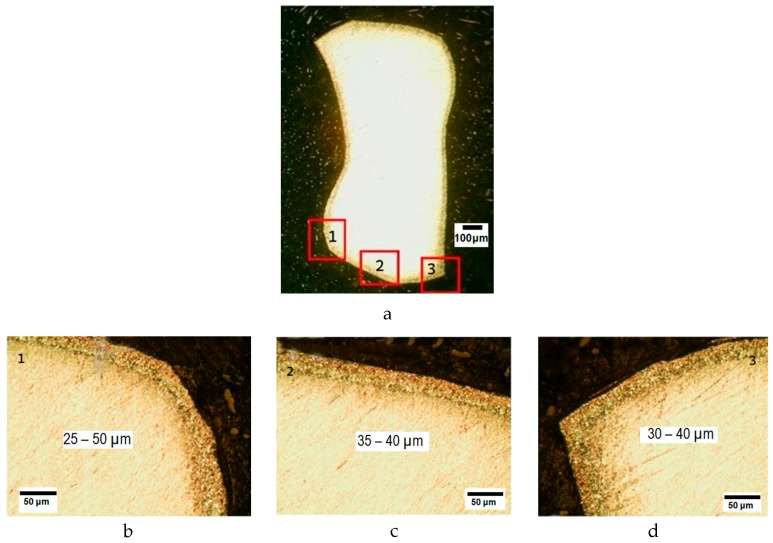
Microstructure of the nitrided layer of the tested sample “drill” made of steel AISI M41 (**a**): microstructure of section 1 of the tested sample (**b**), microstructure of section 2 of the tested sample (**c**), microstructure of section 3 of the tested sample (**d**); 25–50 µm (**b**), 35–40 µm (**c**) and 30–40 µm (**d**)—the thickness of the obtained nitrided layer in sections 1, 2, 3 of the tested sample, respectively.

During the ion nitriding at a temperature of 500 °C for 40 min, a diffusion layer was formed on the surface of drills made of steel AISI M41, the hardness of which was 3–4 times higher than the hardness of the base. The hardness and depth of the nitrided layer in the test samples were characterized as follows: surface hardness 10.0–10.9 GPa, nitrided layer depth based on microhardness 46 μm. The results of the metallographic analysis of M41 steel after ion-plasma nitriding are presented in Figure 10.

**Figure 11:**
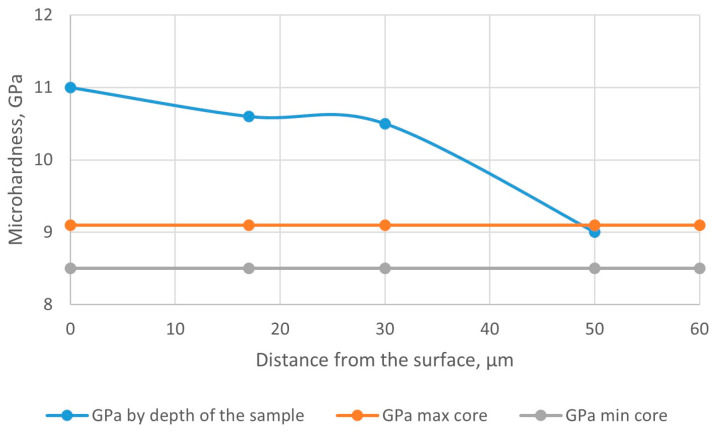
Graph of microhardness distribution by the depth of the nitrided layer on the tested sample “drill” made of steel AISI M41.

The graph of the microhardness distribution along the depth of the nitrided layer on the tested sample “drill” made of steel AISI M41 is shown in Figure 11.

These results are highly significant for industrial applications that require high strength, hardness, and wear resistance. The ion-plasma nitriding process showed considerable potential for enhancing the properties of high-speed steels by forming thin modified layers with improved mechanical and structural characteristics.

### 3.3. Analysis of Wear and Friction of Nitrided Coatings

**Figure 12:**
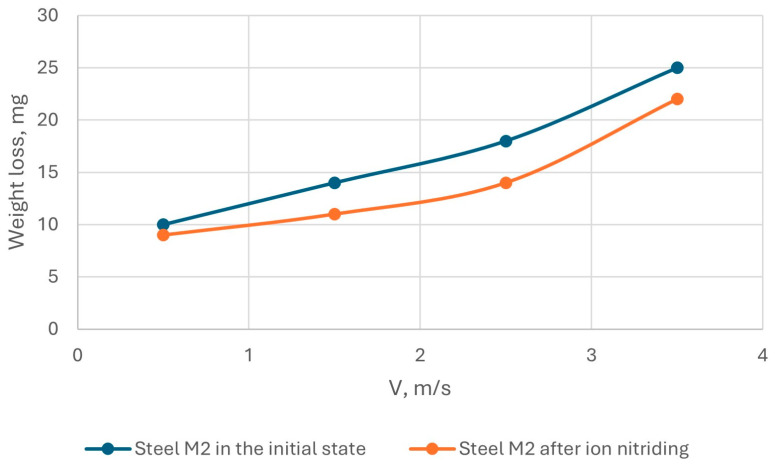
Dependence of the wear rate of AISI M2 steel on the sliding speed.

**Figure 13:**
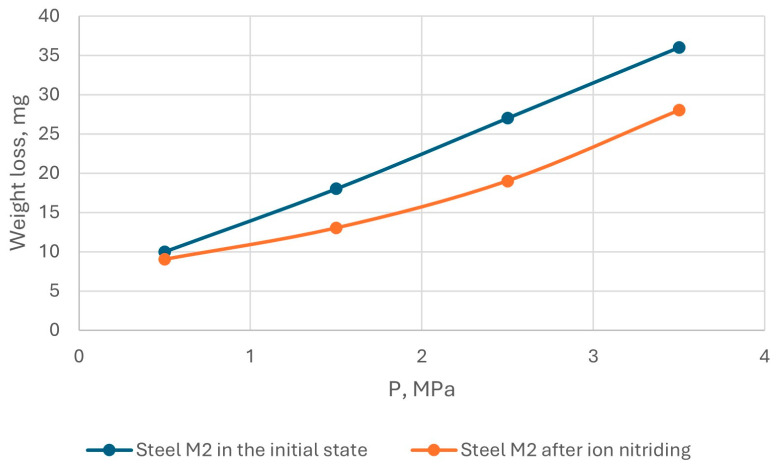
Dependence of the wear rate of AISI M2 steel on the load.

The results of tribological tests of AISI M2 steel in the initial state and after ion-plasma nitriding are shown in Figure 12 and Figure 13.

Tests on the wear resistance of AISI M2 steel under sliding friction conditions without lubrication showed that the wear resistance of steel under these test conditions depends, to a greater extent, on the sliding speed and load: with an increase in the sliding speed, wear increased by almost 1.5 times, and with an increase in the load, wear increased by 2 times. That is, ion-plasma nitriding reduced the wear of AISI M2 steel.

During ion nitriding, the surface hardness and wear resistance of HSSs increase, and the depth of the nitrided layer depends on the phase composition of the steels—with an increase in the amount of carbides in HSS, the depth of the nitrided layer gradually decreases: weaker in steels that do not contain cobalt, more noticeable in cobalt steels.

Wear resistance when cutting with a tool made of nitrided high-speed steel depends on the amount of carbide phase in the steel, increasing as it increases. Thus, to ensure high wear resistance of a tool made of high-speed steel, it is necessary not only to eliminate the presence of excess phases but also to form nitrogen-containing martensite with maximum nitrogen saturation, high compressive stresses, and plastic properties during short-term saturation.

The results of this study demonstrated the significant potential of ion-plasma nitriding for improving the properties of AISI M2 and AISI M41 steels. It was found that increasing the duration of nitriding led to changes in the material structure and the formation of denser layers with high microhardness and corrosion resistance. These results are important for the development of stronger and wear-resistant materials for various industrial applications.

## 4. Discussion

As a result of the conducted research, it was found that the temperature and time of the isothermal holding of the ion nitriding process have a significant impact on the depth of the modified layer of high-speed steels.

Nitrogen diffusion into the depth of steel during ion nitriding is intensified due to three processes: activation of the gas phase, increase in the degree of adsorption, and diffusion coefficient. Positive nitrogen ions in the electrostatic field of the glow discharge acquire a velocity directed normal to the surface of the part. The energy of the ion is 3000 times higher than its energy during furnace nitriding. The temperature and duration of nitriding have a significant effect on the microhardness and rate of nitrogen diffusion.

Based on microscopic and X-ray structural studies, it can be stated that the high hardness of the nitrided layer of high-speed steel is associated with the formation of nitrogenous martensite and γ′-phase, as well as the separation of finely dispersed inclusions of strengthening phases. It should be noted that the formation of a diffusion layer of nitrogen-containing martensite in the surface layers will positively affect the performance of a cutting tool made of high-speed steel. This is because iron nitrides have a higher heat capacity compared to iron. This creates favorable conditions for preventing temperature flares on the surface of the cutting tool.

The research focused on studying the patterns and mechanisms of the formation of thin modified layers in high-speed steel under low-temperature nitriding conditions, as well as on analyzing the influence of the structural and phase state of modified layers on the mechanical, tribological, and physicochemical properties of the material.

In theoretical terms, the mechanism of ion plasma nitriding in steel is based on the process of nitrogen diffusion into the metal. This process includes the activation of the gas phase, an increase in the degree of adsorption, and the diffusion coefficient. The high-energy of nitrogen ions in the electrostatic field of discharge promotes their penetration into the metal, which leads to the formation of a dense dislocation structure in the near-surface layer.

The main components of gas vacuum arc discharge are molecular nitrogen ions N_2_^+^, neutral nitrogen atoms N, and excited nitrogen molecules in various metastable states. Excited nitrogen molecules lose excitation energy upon collision with the sample surface (deactivation of the metastable state occurs), and these molecules do not participate in the nitriding process. Molecular nitrogen ions N_2_^+^ have significant kinetic energy as a result of acceleration in the electric field of the discharge gap and participate in heating the substrate and sputtering its surface, increasing the roughness.

Fe-N compounds have low thermodynamic strength. For example, the energy of formation of the Fe_2_N molecule ∆Gf = 21 kJ/mol, the decomposition temperature is 500 °C; for comparison, the CrN molecule has ∆Gf = 94.3 kJ/mol and 1600 °C, and the Mo_2_N molecule has ∆Gf = 45.7 kJ/mol and 800 °C. Therefore, during bombardment with accelerated particles, simultaneously with the formation of Fe-N compounds, their dissociation occurs, and, thus, the generation of atomic nitrogen is enhanced. A layer of iron nitrides is formed on the surface of the substrate, the ratio of ε- and γ′-phases which depends on the partial pressure of nitrogen, the temperature of the substrate, and the intensity of bombardment (density and energy of the particle flux). With increasing substrate temperature, the dissociation process of Fe-N compounds intensifies, and the proportion of the γ′-phase increases, which, like the ε-phase, disappears with further temperature increase [41,42].

**Figure 14:**
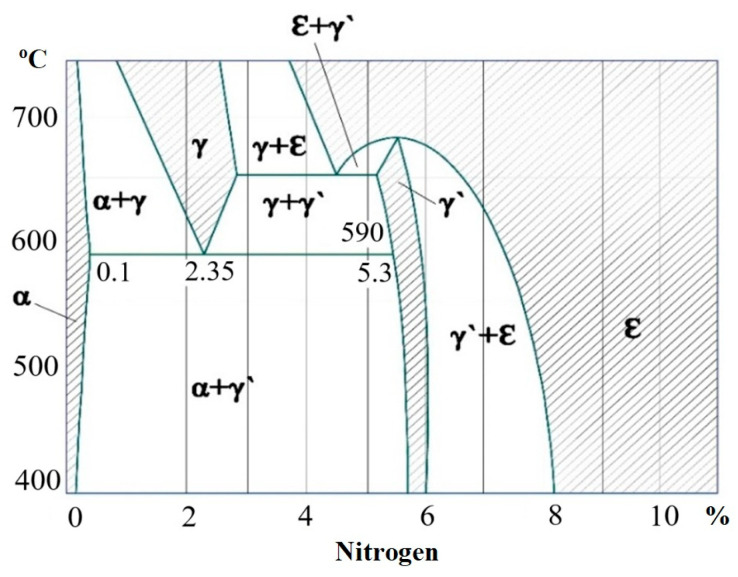
Iron–nitrogen phase diagram; shaded single-phase regions.

Figure 14 shows the Fe-N phase diagram, from which the ε-phase decomposeв at a temperature of about 500 °C and the γ′-phase—at about 700 °C.

According to the diagram of the Fe-N system, the phase composition of the nitrided layer consists of the following zones: the initial structure, where γ’ is Fe_4_N martensite. The results of the analyses indicated that in the surface layer of AISI M2 and M41 steels, which were nitriding, such phrases as Fe_2-3_(N,C), Fe_4_(N,C), (Fe,W)_4_(C,N), CrN were formed [43]. The nitrided layer consists of two main zones: a diffusion sublayer (α), which represents the internal nitrided zone, and a surface martensite zone (ε + γ’), which includes compounds. The ε phase is a solid solution based on Fe_2-3_N martensite, and the γ’ phase is a component of the martensite zone, which consists of a solid solution based on Fe_4_N martensite. During nitriding, alloyed ε and γ’ phases can also formed [41].

The ion-plasma nitriding method makes it possible to obtain layers in which there are practically no iron nitrides, but only a solid solution of nitrogen in it, which is very important for subsequent coating applications. Nitriding conditions allow the change of the parameters of the nitriding process within wide limits and at certain temperatures, pressures, and compositions of process gases to obtain nitrided layers of the required composition. The microhardness of nitrided layers is 10–12 GPa.

The main advantage of ion nitriding of the tool is the possibility of obtaining nitrogenous martensite and dispersed Fe_4_N nitride (γ′-phase) on the surface, unlike classical gas nitriding in ammonia, where the nitride layer consists of two phases—(γ′ + ε), which is a source of internal stresses at the phase interface and causes brittleness and delamination of the hardened layer during operation.

An important factor affecting the nitriding process is temperature. Low temperatures promote the formation of denser structures and deeper nitrogen penetration, which leads to the formation of harder layers. However, it is important to consider that increasing the temperature can cause changes in the structure and properties of the material, which is consistent with [41,42,43,44].

The structural and phase state of the modified layers also affects the mechanical, tribological, and physicochemical properties of the material. The formation of nitrides and other compounds increases the corrosion resistance of the material, making it more resistant to aggressive environments and wear.

Thus, analysis of the results of experimental studies of coatings allows to better understand the mechanisms of formation of thin modified layers in high-speed steels during ion-plasma nitriding. This is an important contribution to the optimization of processing processes and the improvement of material properties for various applications.

## 5. Conclusions

The regularities of the mechanisms of formation of thin modified nitrided layers on the surface of high-speed steels AISI M2 and AISI M41 during ion-plasma nitriding have been investigated.

It was shown that the optimal coating thickness in the range of 25–40 µm was achieved at low temperatures of 480–520 °C during nitriding for up to 1 h. Microstructural analysis revealed a uniform, defect-free coating structure, confirming the high quality of the obtained modified layers.

The surface hardness of AISI M2 and AISI M41 steels after ion-plasma nitriding increased to 10–12 GPa. The high surface hardness of AISI M2 steel is explained by the formation of nitrogen-enriched martensite and fine nitride phases (ε and γ′ phases). The optimal structure of nitrided AISI M41 steel is high-nitrogen martensite without excess nitrides, which provides the required hardness and wear resistance.

By controlling the process parameters, it is possible to adjust the thickness of the modified layer, its phase and structural state, as well as its mechanical properties, which is important in the development of tools made from high-speed steels AISI M2 and AISI M41 operating under various demanding conditions. This makes it possible to use the ion-plasma nitriding method for strengthening heat-treated high-speed steels as a finishing treatment.

## Figures and Tables

**Table 1:** Chemical composition of the studied HSSs, %.

Chemical Element	Mass Fraction of Elements (No more), %	
	Steel AISI M2	Steel AISI M41
Carbon, C	0.82–0.90	0.86–0.94
Silicon, Si	0.20–0.50	0.20–0.50
Chromium, Cr	3.80–4.40	3.80–4.30
Molybdenum, Mo	4.80–5.30	4.80–5.30
Wolfram, W	5.50–6.50	5.70–6.70
Vanadium, V	1.70–2.10	1.70–2.10
Cobalt, Co	up to 0.50	4.70–5.20
Manganese, Mn	0.20–0.50	0.20–0.50
Nikel, Ni	0.60	up to 0.60
Copper, Cu	up to 0.25	up to 0.25
Sulfur, S	up to 0.025	up to 0.03
Phosphorus, P	up to 0.03	up to 0.03
Ferrum, Fe	~80	~75

**Table 2:** Main characteristics of the studied HSSs in the as-delivered condition.

Parameter	Steel AISI M2	Steel AISI M41
Ac1, °C *	815	840
Ar1, °C *	730	765
Forging temperature, °C	1160–850	1160–850
Annealing temperature, °C	840–860	840–860
HB, MPa, no more	2550	2690
Mass fraction of carbide phase, %	22	23

* On the iron–cementite diagram, the critical points forming the PSK line are denoted as Ac1 (during heating) and Ar1 (during cooling). The critical temperatures Ac1 and Ar1 depend on the steel composition. Knowing these critical points facilitates the study of heat treatment processes of steels, as they determine the phase transformations during heating and cooling. Ac1 is the temperature at which pearlite begins to transform into austenite during heating. It defines the minimum temperature for quenching and normalizing steel. Ar1 is the temperature at which austenite begins to transform into pearlite during cooling. It influences the formation of the final steel structure after heat treatment. During quenching, steel is heated above Ac1 to form austenite, which is then rapidly cooled to form martensite. During tempering, the temperature must remain below Ac1 to avoid re-initiation of phase transformation. In normalizing and annealing, heating is performed slightly above Ac1 to obtain a uniform structure.

**Table 3:** Criteria for selection and evaluation of coatings during ion-plasma nitriding.

Evaluation Criteria	Assessment Parameters	Indicative Values
Coating thickness (µm)	Minimum thickness ensuring high durability	5–20 µm
Microstructure	Uniformity, absence of defects	Homogeneous structure without visible defects
Phase composition	Content of different phases, stability	High nitrogen content, stable phases
Mechanical properties	Strength, hardness, elasticity	Strength: 800–1200 MPa, Microhardness: 8–9 GPa
Tribological characteristics	Wear resistance, friction resistance	High resistance to friction and wear

**Table 4:** Properties of samples from steel AISI M2 after ion nitriding for different nitriding times.

Nitriding Time, min	30	40	50	60
Microhardness of the nitrided layer, Gpa	7.10	7.47	8.48	10.50
Depth of nitrided layer, μm	30	40	49	58

## Data Availability

The original contributions presented in this study are included in the article. Further inquiries can be directed to the corresponding author.

## Abbreviations

The following abbreviations are used in this manuscript:

Ac1	The temperature of the beginning of the transformation of pearlite into austenite upon heating
Ar1	The temperature of the beginning of the transformation of pearlite into austenite upon cooling
BCC	Body-centered cubic
HB	Brinell hardness
HRC	Rockwell hardness, C scale
HSS	High-speed steel
M	Metal designation in carbides
